# Sinus bradycardia and acute renal injury secondary to Viperidae snakebite: first case report

**DOI:** 10.3389/fmed.2025.1600067

**Published:** 2025-08-04

**Authors:** Elías David Guamán-Charco, Anabel Paredes-Ponce, Hugo Orellana-Chimbay, Nelson Omares, Genesis Guamán-Charco, Mario Rubio, Jorge Vasconez-Gonzalez, Esteban Ortiz-Prado

**Affiliations:** ^1^One Health Research Group, Faculty of Health Science, Universidad de Las Americas, Quito, Ecuador; ^2^Emergency Department, Marco Vinicio Iza Hospital, Nueva Loja, Ecuador; ^3^Internal Medicine Department, Marco Vinicio Iza Hospital, Nueva Loja, Ecuador; ^4^Faculty of Medicine, University of Guayaquil, Guayaquil, Ecuador; ^5^Cardiology Service, Baca Ortiz Hospital, Quito, Ecuador

**Keywords:** snakebite, sinus bradycardia, acute kidney injury, Viperidae, snakebite envenomation

## Abstract

**Background:**

Worldwide, millions of people suffer from snakebites every year. In Ecuador, as of epidemiological week 30 of 2024, approximately 271 cases have been reported.

**Clinical case:**

A 54-year-old male patient suffered a snakebite from the Viperidae family 24 h ago, on his right upper limb. Classified as moderate envenomation, he was given antivenom and admitted to the hospital. During his stay, he began to show clinical and paraclinical alterations, including sinus bradycardia on the electrocardiogram and acute renal injury, requiring dialysis therapy sessions. In daily ECG controls on day 13, the heart rate normalized. However, after day 22, he was discharged but remained under triweekly dialysis therapy.

**Conclusion:**

Complications from snakebites are rare, both cardiovascular and renal, but can be potentially fatal without early detection and timely treatment.

## 1 Introduction

According to the World Health Organization (WHO), it is estimated that between 4.5 and 5.4 million people are bitten by snakes each year, of which between 1.8 and 2.7 million develop clinical symptoms, and between 81,000 and 138,000 die from complications (1). Several acute systemic syndromes have been described, including coagulopathy with hemorrhagic effects, neurotoxicity, myotoxicity, nephrotoxicity, and cardiotoxicity (2). The venom of the snake *Lachesis acrochorda* has been described to induce blood coagulation by promoting platelet aggregation. In contrast, envenomation by *Bothrops jararaca* causes hemostatic disturbances characterized by ecchymosis, petechiae, purpura, epistaxis, and gingival bleeding (3, 4).

In Ecuador, according to official data obtained from the National Institute of Statistics and Census (INEC), a mortality rate of 0.07 (range: 0.03–0.10) per 100,000 inhabitants was recorded during the period from 2014 to 2019. In addition, according to SIVE-ALERTA-MSP, during the period from 2016 to 2020, 7,569 cases were recorded, with an annual average of 1,514 cases (5). By 2023, 1,407 cases of snakebites had been recorded, and by epidemiological week 30 of 2024, 271 cases had been reported (6). The region with the highest incidence rate is the Amazon, with 55–78 cases per 100,000 inhabitants. A study carried out in the northern Ecuadorian Amazon, during the period from 2017 to 2021, identified 147 cases (29.4 per year), of which 20 (13.6%) were mild, 90 (61.2%) were moderate, and 37 (25.2%) were severe (5).

The snakes of primary toxicological interest identified in Ecuadorian territory include 17 species from the Viperidae family and 18 species from the Elapidae family. In western Ecuador, the Viperidae family includes the species *Bothriechis schlegelii* (parrot parrot), *Bothrops asper* (equis), and *Lachesis acrochorda* (verrugosa); and in the Elapidae family, *Micrurus mipartitus decussatus* (coral) is present (7). In the Ecuadorian Amazon, the Viperidae family includes *Bothriopsis bilineata smaragdina* (lorita machacui, orito machacui, lora), *Bothriopsis taeniata* (shishin), *Bothrocophias hyoprora* (padlock head), *Bothrocophias microphthalmus* (rotten leaf, macanchilla), *Bothrops atrox* (equis, pitalala), and *Lachesis muta* (verrugosa, yamunga); and in the Elapidae family, *Micrurus helleri* (coral) is present (7).

**Figure 1:**
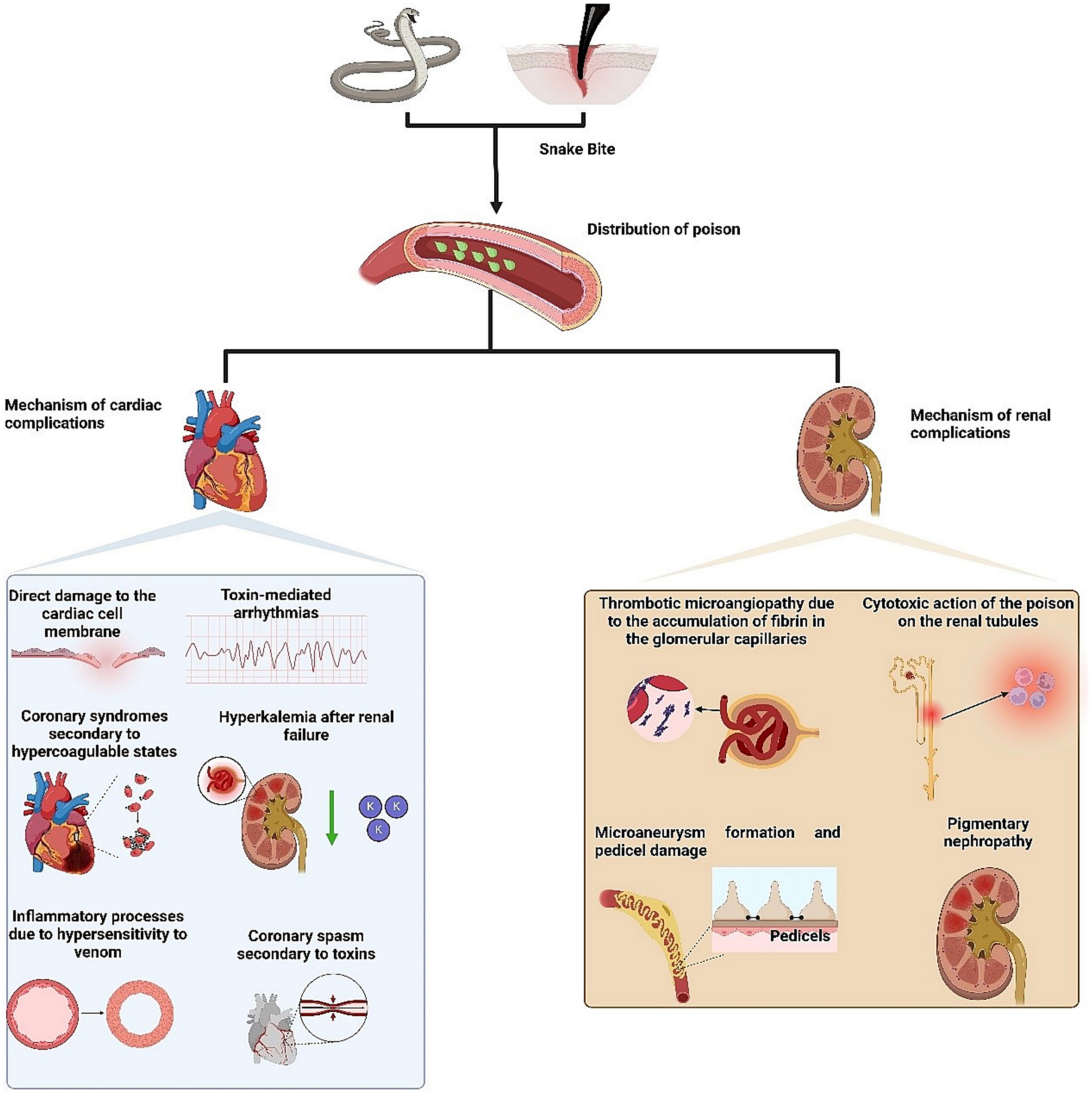
Mechanism of action of snakes venom to cause cardiac and renal complications.

Cardiac complications are rare but can be fatal if not detected in time. Among the described cardiac complications are bradycardia or tachycardia, cardiac arrhythmias, hypertension, atrioventricular block, QT interval alterations, ST segment elevation, T wave inversion, acute myocardial infarction, hypotension, bundle branch block, myocarditis, cardiac arrest, Takotsubo cardiomyopathy, pulmonary hypertension, cardiogenic shock, and heart failure (8–11). Kim et al., in their review, described the presence of cardiovascular events in 13.8% of patients. They reported myocardial injury in 13.8% of patients, elevated hs-TnI levels in 10.8%, ischemic changes detected by ECG in 3.1%, and shock in 3.1% of patients (12). Isbister et al., for their part, reported the presence of cardiovascular collapse in 157 individuals who had been bitten by snakes (13). The snake families involved in cardiac complications include Viperidae (e.g., envenomation by *Daboia russelii* has been described to cause atrial fibrillation), Elapidae (*Bungarus cf. sindanus* has been reported to induce ventricular tachycardia and impaired left ventricular systolic function, while *Bungarus caeruleus* can lead to the development of cardiogenic pulmonary edema), Colubridae (e.g., bites from *Ahaetulla nasuta* have been associated with biphasic T wave inversions), and Lamprophiidae (*Atractaspis* spp. envenomation may cause precordial pain, bradycardia, cardiac arrhythmia, and hypertension) (14–18). The pathophysiology of cardiovascular implications in snakebites is poorly understood, although several mechanisms have been hypothesized (Figure 1): (1) direct damage to the cardiac cell membrane, (2) toxin-mediated arrhythmias, (3) coronary syndromes secondary to hypercoagulable states, (4) coronary spasm secondary to toxins, (5) hyperkalemia following renal failure, and (6) inflammatory processes due to hypersensitivity to venom (9).

On the other hand, within renal complications, acute kidney injury (AKI) is a known potentially fatal systemic effect of snake envenomation, especially from the Viperidae and Elapidae families (2). The incidence of AKI ranges from 8 to 60%, with between 15 and 92% of patients requiring some form of renal replacement therapy, and an overall mortality rate of up to 45% (2). Additionally, it has been observed that between 8 and 50% of patients with AKI develop chronic kidney disease. Several pathophysiological mechanisms have been proposed to explain snakebite-induced renal injury, including (Figure 1): (1) thrombotic microangiopathy due to the accumulation of fibrin in the glomerular capillaries, (2) cytotoxic action of the venom on renal tubules mediated by various cytokines, adhesion molecules, complement activation, and release of free radicals, (3) formation of microaneurysms and damage to the podocytes mediated by the proteolytic activity of the venom, and (4) pigmentary nephropathy triggered by phospholipase A2, which leads to rhabdomyolysis and myopathy (19).

The following article aims to describe the first case report of sinus bradycardia and acute kidney injury following a Viperidae snakebite in an Ecuadorian male patient who required permanent dialysis therapy at the Marco Vinicio Iza General Hospital, located in the northern Ecuadorian Amazon.

## 2 Case report

The case of a 54-year-old male patient is described. He lives in a rural area; is mestizo. He has a medical history of type II diabetes mellitus and hypertension (HTA) without medical treatment. He presented to the emergency department, reporting that approximately 24 h ago, he was bitten by a snake identified as a “lora” on his right shoulder while walking on an Amazon trail. The geographic location of the snakebite was Parroquia 10 de Agosto, north of Lago Agrio Canton, in the province of Sucumbíos. It is approximately 19 km from Hospital Marco Vinicio Iza. He complained of moderate pain at the affected site, with a Visual Analog Scale (VAS) score of 7/10, without any other accompanying symptoms.

**Figure 2:**
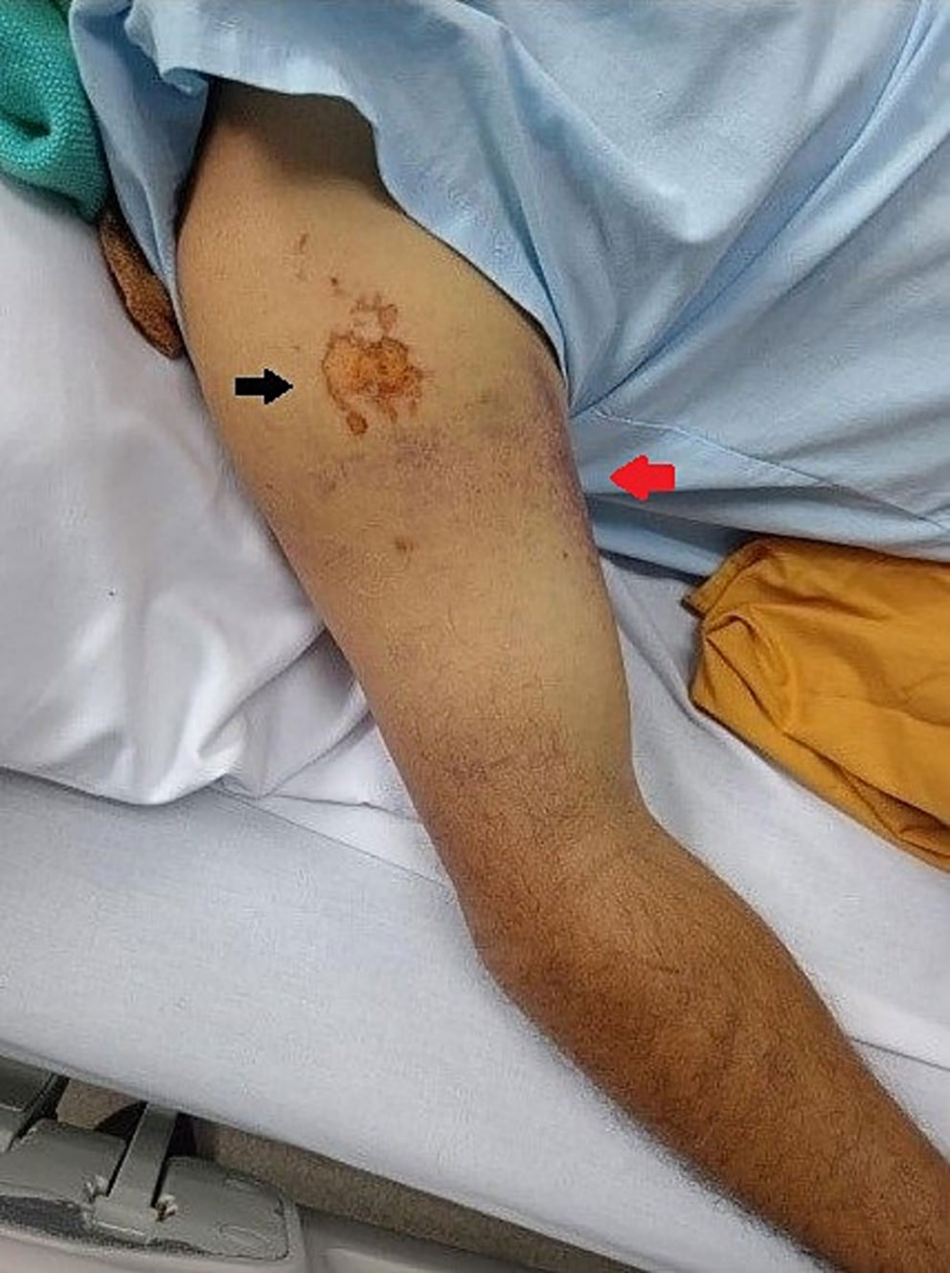
Anatomical site of snake bite. The black arrow indicates the site of the bite, and the red arrow points to the ecchymosis.

On physical examination, the following vital signs were noted upon admission: BP 130/77 mmHg, HR 54 bpm (before the snakebite, the patient had normal HR. According to the Health Care Registration Platform System (PRASS), the heart rate data were as follows: 74 bpm on 26/09/2023 and 84 bpm on 27/09/2023.), RR 20 rpm, T° 36.7°C, SatO2 98%. Anthropometric measurements: Weight 82 kg, Height 1.68 meters. The regional examination revealed blisters and edema (++/+++) at the shoulder level of the right upper limb, extending to the middle third of the forearm (two segments). Distal pulses were present (Figure 2).

A capillary blood glucose test was performed, resulting in 112 mg/dL, and a coagulation test was positive (no coagulation after 20 min). The patient was classified as having a moderate toxic effect from snake venom, and 8 vials of antivenom were prescribed initially. During the administration of antivenom, the patient did not experience any adverse reactions. The antivenom used was a lyophilized polyvalent antivenom produced by the Clodomiro Picado Institute in Costa Rica. Each vial contains less than 1.2 g of equine immunoglobulins. Each 10 mL vial is capable of neutralizing no less than 30 mg of *Bothrops asper* venom, 20 mg of *Crotalus simus* venom, 30 mg of *Lachesis stenophrys* venom, and 30 mg of *Bothrops atrox* venom.

Admission paraclinical tests showed the following results: Leukocytes: 12.05, Neutrophils: 74.7%, Lymphocytes: 14.8%, Monocytes: 10%, Hemoglobin: 13.2 g/dL, Hematocrit: 40.9%, Platelets: 100,000, PT: 13.8, PTT: 55.2, INR: 1.7, Glucose: 109 mg/dL, Urea: 83 mg/dL, Creatinine: 5.2 mg/dL, Electrolytes: (Sodium 135.94, Potassium 3.85, Chlorine 100.08).

**Figure 3:**
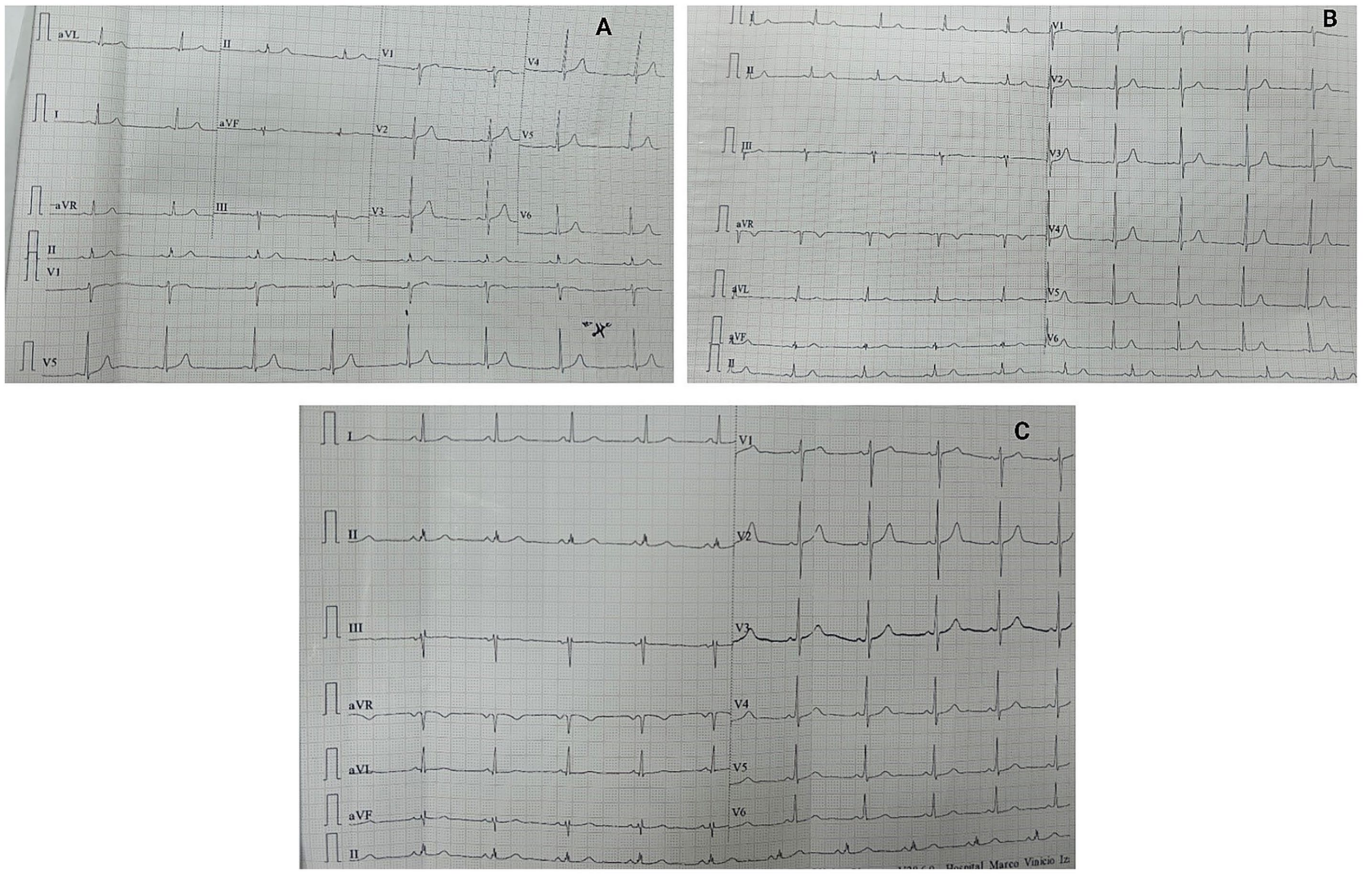
ECG performed on the patient. **(A)** Admission electrocardiogram, showing sinus bradycardia with a heart rate of 47 beats per minute. **(B)** ECG performed on the sixth day of hospital stay showed persistent sinus bradycardia with a heart rate of 56 beats per minute. **(C)** ECG performed on the thirteenth day of hospital stay, sinus rhythm was evident with a heart rate of 63 beats per minute.

An electrocardiogram (ECG) was performed, which showed sinus bradycardia (Figure 3A). Twenty-four hours after admission, an abdominal ultrasound was conducted, revealing a simple renal cyst in the right kidney with no other abnormalities.

**Figure 4:**
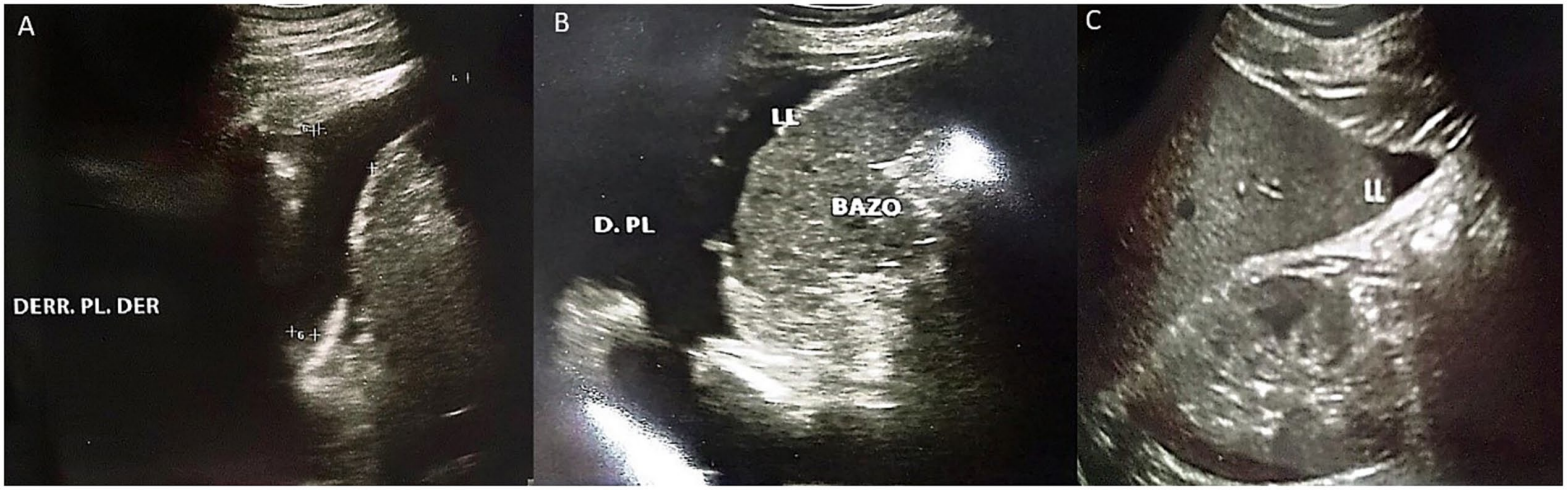
Abdominal ultrasound performed on the third day of hospitalization. **(A)** Presence of right pleural effusion is evident. **(B)** Presence of left pleural effusion is evident, as well as free fluid bordering the spleen. **(C)** Presence of free fluid bordering the liver.

On the second day of hospitalization, the patient progressively developed elevated blood pressure, an icteric tint, and abdominal distension. Complementary studies revealed thrombocytopenia, prolonged coagulation times, severe anemia, and increased nitrogen levels, requiring transfusions of blood products (fresh frozen plasma, red blood cell concentrate, and platelets). Tests for dengue and hematogenous organisms were negative (Table 1). A follow-up abdominal ultrasound on the third day revealed bilateral pleural effusion and ascites (Figure 4). At the sixth day the ECG continued to show persistent sinus bradycardia (Figure 3B).

**Table 1:** Laboratory tests during hospital stay.

Data	Value																	Reference value
	Admission	Day 1	Day 2	Day 3	Day 4	Day 5	Day 7	Day 8	Day 9	Day 10	Day 11	Day 13	Day 14	Day 15	Day 18	Day 19	Day 20	
Complete blood count																		
Leukocytes	12.05	10.39	8.69	7.86	13.32	10.1	12.25	12.98	12.54	14.01	15.87	9.66			8.44	8.86	8.36	5.00–15.00 × 10^3^/uL
Neutrophils	74.7	70.3	74.5	78.9	90.3	91.1	85.6	73.3	76.3	77.9	83.3	74.4			77.9	72.6	71.5	46–62%
Lymphocytes	14.8	21.4	14.7	18.8	5	5.3	8.9	19	16.2	15.6	12.3	17.7			12.5	14.8	16.9	28–44%
Eosinophils	0.1	1.6	1.1	1.0	0	0	0	0.2	0.5	0.7	0.2	2.8			2.2	2.6	4.1	1–6%
Platelets	100	46	30	30	35	46	94	161	154	187	247	318			218	181	171	150–450 × 10^3^/uL
Red Blood Cells	4.35	3.83	3.42	3.31	2.57	2.48	2.4	2.35	2.55	3.58	3.65	3.84			3.61	3.36	3.54	4.30–5.70 × 10^3^/uL
Hemoglobin	13.2	11.4	10	9.7	7.8	7.3	7.3	6.9	7.7	10.5	10.7	11.5			10.6	10.3	10.4	13.20–17.80 g/dL
Blood chemistry																		
Glucose		97	82		148	141												
Urea	83	108	137		229	221	450	209		215	159	183			141	164	159	10–48.5 mg/dL
Creatinine	5.2	7.3	8.6		11.7	10.7	13.3	9.1		8.1	6	6.2			4.7	5	4.6	0.80–1.30 mg/dL
Uric acid			6.1															3.6–8.20 mg/dL
Total cholesterol			109															120–200mg/dL
AST		117.23	88.07		45.82	32.92		18.57	15.41									0.00–35.00 U/L
ALT		65.28	59.50		52.35	42.38		31.22	20.35									0.00–45.00 U/L
Total bilirubin		8.13	7.47	7.68	3.84	2.32		1.51	1.09									0.30–1.10 mg/dL
Direct bilirubin		2.63	2.95	4.11	1.84	1.08		0.79	0.51									0.10–0.40 mg/dL
Indirect bilirubin		5.50	4.52	3.57	2	1.24		0.72	0.58									0–0.90 mg/dL
GGT		25.69	37.78					39.87										11–61 U/L
Amylase		105.63	108.02															28–100 U/L
Lipase		30.81	36.14															0–60 U/L
Total proteins								5.02		3.64								6.60–8.70 g/dL
Albumin								3.33		3.37								3.97–4.94 g/Dl
IgM Dengue		0.70 (negative)																Positive >1.1 Indeterminate 0.9–1.0 Negative < 0.9
Dengue NS-1		0.86 (negative)																Positive >1.1 Indeterminate 0.9–1.0 Negative < 0.9
Electrolytes																		
Na	135.94	135.96	138.61		132.32	135.95				142.11	139.94	144.70						135–145 mmoL/mL
K	3.85	4	3.84		4.96	4.62				3.59	3.80	4.11						3.50–5.50 mmoL/mL
Cl	100.08	105.41	108.03		105.47	104.19				105.22	104.68	104.42						96–100 mmoL/mL
Cardiac enzymes																		
CK-MB											3.9							3–100 ng/mL
Troponi I											0.35							0.01–15 ng/mL
Coagulation times																		
PT	13.8	16.5	15.7	15.5		13.6	15.6	63.3	14.1	13.8	12.6		13.9	16.0		15.5	15	9.5–14 s
APTT	55.2	42.7	44.7	43		33.8	35.9	Does not clot	30.2	31.1	34.3		28.2	34.6		36.7	38.8	22–40 s
INR	1.7	1.4	1.3	1.3		1.2	1.4	6.3	1.2	1.1	1.1		1.2	1.4		1.3	1.3	0.85–1.30
D-Dimere											15.03	11.64						<1.0

Vital signs																			
	Admis sion	Day 1	Day 2	Da y 3	Day 4	Day 5	Da y 7	Day 8	Da y 9	Day 10	Day 11	Day 13	Day 14	Day 15	Day 18	Day 19	Day 20	Day 21	Day 22
Systolic pressure mmHg	130	116	115	123	122	167	107	107	115	157	164	139	113	139	100	138	135	110	106
Diastolic pressure mmHg	77	74	70	74	79	97	64	57	68	81	89	100	70	81	64	74	67	63	59
Heart rate	80	52	56	58	56	58	54	56	62	64	48	54	58	56	72	72	70	72	70

Given the clinical deterioration and the paraclinical results, the patient required 8 sessions of dialysis therapy, with subsequent controls showing a decrease in nitrogen levels. He was also monitored with daily ECGs, which showed persistent sinus bradycardia until day 13, when the heart rate normalized to greater than 63 beats per minute (Figure 3C).

The patient was hospitalized for 22 days. Upon discharge, he was prescribed antihypertensive medication (nifedipine 10 mg every 12 h) and continued follow-up with nephrology, receiving dialysis every 3 weeks.

## 3 Discussion

Cardiovascular complications, especially sinus bradycardia, are rare, as is acute kidney injury, which in many cases requires temporary and/or definitive dialysis therapy. Early detection poses a challenge for healthcare personnel, considering that most snakebite incidents occur in rural areas with limited infrastructure and medical resources, making it difficult to collect detailed data (9, 19). In the present case, the patient developed thrombocytopenia, which is one of the main hematotoxic effects of snake venom. This condition is often secondary to disseminated intravascular coagulation (DIC) and venom-induced coagulopathy (20, 21). Anemia was also observed. Snakebite envenomation has been reported to cause consumption coagulopathy, and some patients may develop thrombotic microangiopathy, which manifests as microangiopathic hemolytic anemia (22). Moreover, the association between microangiopathic hemolytic anemia, acute kidney injury, and thrombocytopenia with venom-induced consumption coagulopathy has been recognized (23).

### 3.1 Sinus bradycardia after snakebite

With respect to the type of snake, cases of bradycardia have been reported in snakes from the Viperidae family (24). In this case report, the patient identified the species *Bothriopsis bilineata smaragdina*, known as “lora,” which belongs to the Viperidae family. It is important to highlight that in the patient, myocardial injury markers (troponin I and CK-MB) were normal, but there was cardiac alteration (sinus bradycardia). Studies in animal models have indicated that the negative inotropic effect induced by the venom is not related to cardiac toxicity and occurs through the NO/cGMP/PKG pathway. This was observed in a study analyzing the venom of *Crotalus durissus cascavella* in rats (25).

A study conducted by Nayak et al. in 30 patients observed cardiotoxicity in 25% of cases due to Viperidae snakes; among these, bradycardia was observed in 10% (26). ECG studies in patients with envenomation often show nonspecific changes, such as sinus arrest with junctional escape rhythm and retrograde P waves, with a heart rate of 40 beats/min, suggestive of sinus node dysfunction. However, on the third day of hospitalization for snake envenomation, a repeat ECG showed normal sinus rhythm (10). In another case report, sinus bradycardia followed by pulseless electrical activity was evident, requiring two rounds of advanced cardiac life support before return of spontaneous circulation (27). A possible direct effect of the venom on the atrioventricular node has been considered as a cause of bradycardia (24). Additionally, it has been proposed that bradycardia may occur due to parasympathetic stimulation, possibly induced by fear (11).

Different components of snake venom can affect heart rate. Bradykinin-potentiating peptides decrease the concentration of angiotensin II, leading to a reduction in blood pressure. Additionally, these peptides increase bradykinin levels, which have vasodilatory properties and can also act on receptors in the nucleus ambiguus to induce bradycardia (28, 29). On the other hand, beta-cardiotoxin, a three-finger toxin, exhibits *β*-blocker activity, as evidenced by its negative chronotropic effects and its ability to bind to β1- and β2-adrenergic receptors, causing bradycardia (30, 31). Cysteine-rich secretory proteins have also been reported to exert non-enzymatic inhibitory activity on L-type Ca^2+^ channels, leading to the development of bradycardia (32, 33). Furthermore, natriuretic peptides present in various snake species promote vascular relaxation and reduce myocardial contractility (28).

Studies using venom from various snake species have been conducted in animal models. Simões et al. analyzed the influence of *Crotalus durissus cascavella* venom on cardiac activity in rats. The results revealed that the venom induced hypotension and bradycardia, and they noted that the venom-induced negative inotropic effect occurs through the NO/cGMP/PKG signaling pathway, which likely leads to both hypotension and bradycardia (25). Similarly, Angel-Camilo et al. observed comparable findings in their studies analyzing the effects of *Lachesis acrochorda* venom on cardiovascular parameters in rats. Their results showed that the venom induced blood coagulation, platelet aggregation, hypotension, and bradycardia, in addition to increased P and T waves, prolonged QT interval, and elevated serum CK and CK-MB levels (3, 34).

In a study analyzing 80 cases of snakebite, bradycardia was observed in 10 cases (18.5%) (35). Greene et al. reported the case of a patient who was bitten by a *Naja kaouthia* cobra. The patient developed mild hypotension and transient bradycardia, and upon being admitted to the intensive care unit, experienced another episode of bradycardia that was corrected with atropine (36). Sachett et al. reported the case of a 65-year-old patient who was bitten by a *Bothrops atrox* snake, which led to the development of bradycardia (37).

Sartim et al., in their study evaluating plasma markers in 80 patients who had suffered bites from *Bothrops atrox*, observed elevated levels of FABP3 in at least 98.7% of the patients, troponin I in 12.5%, and CK-MB in 8.8%. Additionally, plasma levels of fibrin/fibrinogen degradation product, tissue factor, and factor VII were positively correlated with troponin I concentrations, leading to the conclusion that myocardial injury is directly associated with venom-induced coagulopathy (38).

Additionally, it is important to highlight endothelial dysfunction, particularly in the coronary vasculature. This dysfunction can be triggered by the inflammatory response induced by various components present in snake venom. Among them, phospholipase A₂ (PLA₂) stands out, as it promotes the rupture of the cell membrane, leading to the release of arachidonic acid (39). This, in turn, contributes to the production of PGE₂, leukotrienes, lipoxins, and epoxyeicosatrienoic acids, which can initiate the inflammatory (39). Metalloproteinases, on the other hand, stimulate the release of inflammatory mediators such as ICAM-1, IL-8, TNF-*α*, and MCP-1 from endothelial cells, leading to apoptosis. Meanwhile, cysteine-rich secretory protein increases the expression of IL-6 and plays a role in sustaining inflammation (39). Snake venom can also directly affect endothelial cells by delaminating the endothelial monolayer from the underlying matrix and disrupting endothelial cell membranes (40). Another important mechanism to highlight regarding snake venom is the production of reactive oxygen species, which, when accumulated, induce oxidative stress. Free radicals can damage cellular DNA, leading to cell dysfunction, cell cycle arrest, and cell death. Hydrogen peroxide has been shown to increase the activity of caspases and pro-apoptotic enzymes, cause the loss of mitochondrial membrane potential, and degrade DNA (41–43).

Regarding treatment, this typically includes anti-venom therapy, hemodynamic support, and treatment of the specific cause if applicable. It has been suggested that anti-venom therapy alone may be sufficient for snakebites with cardiac involvement. According to Bhatt et al., in their case report, a resolution of bundle branch block following snakebite was evident with anti-venom therapy (44).

### 3.2 Acute kidney injury after snakebite

At the renal level, histopathological changes secondary to a snakebite have been observed, including acute cortical necrosis, thrombotic microangiopathy, diffuse mesangial proliferation, isolated glomerular thrombosis, acute tubular necrosis, acute interstitial nephritis, pigment-induced nephropathy, necrotizing arteritis, and thrombophlebitis (45). An observational study of 184 patients following a hemotoxic snakebite demonstrated higher mortality rates in patients with stage 3 AKI, according to the KDIGO (Kidney Disease: Improving Global Outcomes) scale [relative risk (RR) 4.45 (95% CI 1.14–17.42)] (46). Additionally, a study conducted at the HGMVI showed that out of 147 patients who suffered a snakebite, 2 patients developed acute kidney injury (5).

Among the factors associated with the development of AKI, the following have been identified: age, body surface area, the age of the snake, amount of venom inoculated, potency and composition of the venom, bite location, time between the accident and the administration of anti-venom, hospitalization duration, hypotension, prolonged coagulation times, anemia, hyperbilirubinemia, elevated LDH, hypoalbuminemia, leukocytosis, and disseminated intravascular coagulation (47, 48). Furthermore, the presence of comorbidities increases the susceptibility of patients to the venom, including arterial hypertension, diabetes mellitus, coronary artery disease, and underlying kidney disease (47). This patient, in particular, was identified as having hypertension and diabetes mellitus, which were not under medical treatment.

On the other hand, it is important to highlight two concepts. The first is the cardiorenal syndrome, which encompasses a wide range of interrelated disorders that may be acute or chronic, with the heart or kidneys as the primary affected organs, thus emphasizing the bidirectional nature of cardiorenal interactions (49). Both type 1 and type 2 cardiorenal syndromes lead to kidney injury. This occurs because, when fluid overload develops as a result of worsening cardiac function, venous pressure increases and is transmitted to the efferent arterioles, causing a decrease in glomerular filtration pressure and kidney injury (50). Moreover, when cardiac output and mean arterial pressure decrease, renal blood flow is reduced, which activates the renin–angiotensin system, reduces nitric oxide in the endothelium, activates the sympathetic nervous system, and induces inflammatory mediators. All of these factors cause structural and functional damage to both the kidneys and the heart (51). In addition, oxidative stress triggers the release of IL-6, IL-1, and tumor necrosis factor-alpha, impairing renal compensatory mechanisms (52).

The second is the cardiovascular-renal-metabolic syndrome, a disorder attributable to the relationship between obesity, diabetes, chronic kidney disease, and cardiovascular diseases (57). It has been described that when diabetes mellitus and hypertension coexist (as in the case of this patient with untreated type 2 diabetes mellitus and hypertension), their impact on the kidneys becomes even more severe (53). This is because hyperglycemia stimulates the production of reactive oxygen species, leading to oxidative stress and the formation of advanced glycation end products (AGEs). Moreover, AGEs interact with receptors for advanced glycation end products on vascular and renal cells, contributing to tissue stiffening and the activation of pro-inflammatory and pro-fibrotic mechanisms (53).

It is estimated that approximately one in five patients with acute heart disease has experienced acute kidney injury or worsening of renal function. Moreover, cardiovascular disease accounts for more than half of all deaths in patients with cardiorenal syndrome—10 to 20 times more than in individuals of the same age without this syndrome (52, 54). The management of cardiorenal syndrome focuses on addressing the underlying etiology and improving the syndrome’s complications. In individuals who exhibit a positive diuretic response, every effort should be made to achieve fluid removal using diuretics, either through continuous infusion or intravenous bolus administration (50, 54). Additionally, inotropic agents can be used in refractory cases and may even help improve cardiac function and reduce venous congestion (50). In the case of cardiorenal metabolic syndrome, treatment includes lifestyle modifications, dietary intervention, and increased physical activity aimed at achieving weight loss. In addition to addressing comorbidities associated with cardiovascular, renal, and metabolic conditions, the use of SGLT2 inhibitors and GLP-1 receptor agonists has been shown to be effective in patients with type 2 diabetes mellitus (55). Optimal blood pressure levels (<130/80 mmHg) should also be maintained in these patients, for which ACE inhibitors or angiotensin II receptor blockers (ARBs) can be used (55).

The treatment for managing acute kidney injury after a snakebite is based on anti-venom therapy, along with the reduction of nitrogen and hyperkalemia, which in most cases requires dialysis therapy (56). Additional measures include maintaining adequate blood pressure levels and avoiding compartment syndrome, the latter due to the risk of metabolic acidosis and rhabdomyolysis (2, 19).

## 4 Conclusion

Complications from snakebites, both cardiovascular and renal, are rare but potentially fatal without early detection and timely treatment. Additionally, it is important to note that factors such as the patient’s underlying diseases contribute to worsening the complications caused by the snakebite, and in this case, the patient’s conditions were even untreated. Early administration of antivenom remains the cornerstone of treatment, along with comprehensive and multidisciplinary monitoring and management of complications, which, as observed in this case report, occurred simultaneously.

## Data Availability

The original contributions presented in the study are included in the article/supplementary material, further inquiries can be directed to the corresponding author.
